# Bacteriophage-Mediated Control of Biofilm: A Promising New Dawn for the Future

**DOI:** 10.3389/fmicb.2022.825828

**Published:** 2022-04-04

**Authors:** Cheng Chang, Xinbo Yu, Wennan Guo, Chaoyi Guo, Xiaokui Guo, Qingtian Li, Yongzhang Zhu

**Affiliations:** ^1^School of Global Health, Chinese Center for Tropical Diseases Research, Shanghai Jiao Tong University School of Medicine, One Health Center, Shanghai Jiao Tong University-The University of Edinburgh, Shanghai, China; ^2^College of Stomatology, Shanghai Jiao Tong University School of Medicine, Shanghai, China; ^3^Department of Laboratory Medicine, Ruijin Hospital, Shanghai Jiao Tong University School of Medicine, Shanghai, China

**Keywords:** bacteriophages, phage therapy, biofilms, depolymerase, endolysin

## Abstract

Biofilms are complex microbial microcolonies consisting of planktonic and dormant bacteria bound to a surface. The bacterial cells within the biofilm are embedded within the extracellular polymeric substance (EPS) consisting mainly of exopolysaccharides, secreted proteins, lipids, and extracellular DNA. This structural matrix poses a major challenge against common treatment options due to its extensive antibiotic-resistant properties. Because biofilms are so recalcitrant to antibiotics, they pose a unique challenge to patients in a nosocomial setting, mainly linked to lower respiratory, urinary tract, and surgical wound infections as well as the medical devices used during treatment. Another unique property of biofilm is its ability to adhere to both biological and man-made surfaces, allowing growth on human tissues and organs, hospital tools, and medical devices, etc. Based on prior understanding of bacteriophage structure, mechanisms, and its effects on bacteria eradication, leading research has been conducted on the effects of phages and its individual proteins on biofilm and its role in overall biofilm removal while also revealing the obstacles this form of treatment currently have. The expansion in the phage host-species range is one that urges for improvement and is the focus for future studies. This review aims to demonstrate the advantages and challenges of bacteriophage and its components on biofilm removal, as well as potential usage of phage cocktail, combination therapy, and genetically modified phages in a clinical setting.

## Introduction

**FIGURE 1:**
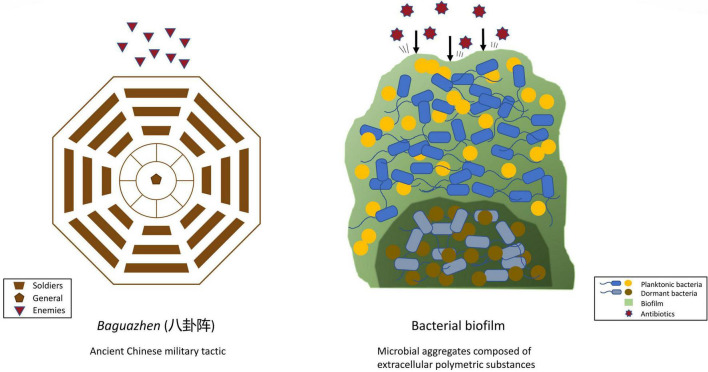
Comparing the structural mechanism of ancient Chinese military tactics and bacterial biofilm.

Ancient Chinese warfare often had soldiers, in an arranged formation, form a fortress-like circle to shield themselves from and strike their opponents, as depicted in Figure 1. Similarly, bacteria benefit from working in a group by building a protective system that individual bacteria find difficult to achieve. Working together also allows bacteria to effectively conduct a collective assault on the host’s defense or immune system. Having a medium to host a community of bacteria, therefore, is vital, and is achieved by the formation of biofilm. Biofilm is a microbial aggregate composed of an extracellular polymeric substance matrix secreted by the microbes themselves.

The topic of biofilm raises global health concerns as the presence of biofilm is responsible for the majority of bacterial infections due to its ability to promote microbial survival against external stimuli, including antibiotics (Harrison et al., 2007; Høiby et al., 2011; Lebeaux et al., 2013; Wu et al., 2019; Yin et al., 2019). In addition, the presence of biofilm formation on medical devices presents a major challenge for modern medicine, especially on artificial joint restorations and catheters (Morris et al., 2019; Cano et al., 2021).

Hence, new strategies to eliminate biofilm function are in demand and bacteriophage therapy is an option regaining attention in recent decades. Bacteriophages—commonly simplified as phages—are the most abundant micoorganisms on the planet. Phages are viruses that selectively target and specifically kill bacteria through a replication cycle that involves attachment, injection of genetic information, replication within the cell, viral assembly, and—in lytic phages—kill the host bacteria cell by lysing the cell wall. Phages provide researchers and clinicians alike with a new dimension in antibacterial combat.

## What Is Biofilm?

**FIGURE 2:**
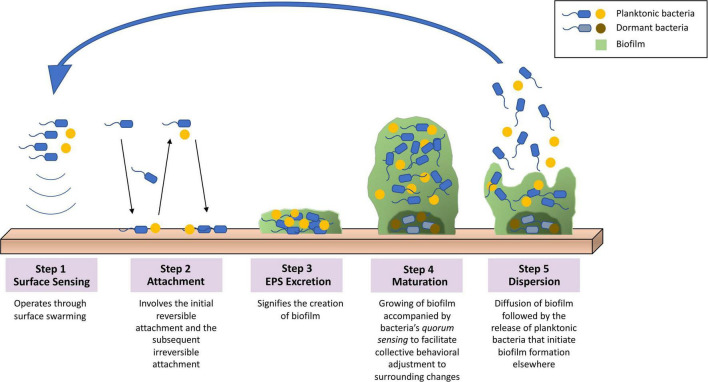
Overview of the biofilm formation process. (1) Surface sensing operated by surface swarming. (2) Attachment stage involving the initial reversible attachment and the subsequent irreversible attachment. (3) Excretion of EPS that signifies the creation of biofilm. (4) Maturation of biofilm that involves quorum sensing to facilitate collective behavioral adjustments to surrounding changes. (5) Dispersal of biofilm structure following the release of planktonic bacteria initiating biofilm formation elsewhere.

The extracellular polymeric substance (EPS) matrix composed in biofilm consists of exopolysaccharides, secreted proteins, lipids, extracellular DNA, and other minor components (Whitchurch et al., 2002; Rabin et al., 2015). The characteristics of the matrix components as well as the interactions of molecules provide mechanisms to biofilm’s adherence to varieties of surfaces, preservation of nutrient reservoirs, and protection from outer environment (Yan and Bassler, 2019). Biofilm is commonly formed on surfaces such as dental deposits and medical implants, while it is also capable of forming without said surfaces (O’Toole et al., 2000; Alhede et al., 2011). The formation of biofilm is a complex yet well-regulated process that can be categorized into five main steps, as demonstrated in Figure 2: (i) surface sensing operated by the planktonic bacteria’s flagella that facilitates such signaling through surface swarming (Armbruster and Parsek, 2018). (ii) attachment stage that involves the initial reversible attachment, responsible for loosely adhering the surface and detaching, and the subsequent irreversible attachment, responsible for more specific and stable adherence that is carried out by the bacterial adhesions (Rabin et al., 2015; Armbruster and Parsek, 2018). (iii) excretion of EPS matrix produced by the recently attached bacteria that signifies the creation of biofilm (Rabin et al., 2015). (iv) maturation of biofilm that involves interactions between bacteria cells that leads to the formation of microcolonies (Muhammad et al., 2020). (v) dispersal of biofilm structure following the release of planktonic bacteria and initiate the formation of biofilm at other sites (Rabin et al., 2015).

The adaptation and survival of biofilm are further accomplished via quorum sensing (QS), a communication system between resident bacteria cells that lead to a collective behavioral adjustment to change in cell density or other surrounding conditions (Remy et al., 2018). QS also plays an important role in regulating virulence factors in biofilm and contributing additional defensive mechanisms against foreign stress (Høyland-Kroghsbo et al., 2013).

Biofilm-resident bacteria can be categorized based on its Gram stain (Gram-positive or Gram-negative) or its growth site. The most encountered Gram-positive bacterial species include *Staphylococcus aureus (S. aureus), Listeria monocytogenes (L. monocytogenes), Bacillus subtilis (B. subtilis)*, and *Enterococcus faecalis (E. faecalis)* (Abee et al., 2011; Cruz et al., 2018). Gram-negative bacteria, on the other hand, are more prevalent and clinically significant specifically in the nosocomial setting due to its higher multidrug resistance rate (Spellberg et al., 2013). Frequently isolated Gram-negative bacteria include *Pseudomonas aeruginosa (P. aeruginosa)* (Bisht et al., 2021), *Klebsiella pneumoniae (K. pneumoniae)* (Tabassum et al., 2018), *Acinetobacter baumannii (A. baumannii)* (Shahed-Al-Mahmud et al., 2021), *Escherichia coli (E. coli)* (Sharma et al., 2016), *Proteus mirabilis (P. mirabilis)* (Wasfi et al., 2020), and *Streptococcus pneumoniae (S. pneumoniae)* (van der Kamp et al., 2020). In addition, bacteria and its biofilm could also be classified depending on whether a foreign body is involved or not. Common bacteria that cause infection through the formation of biofilm on medical devices include *P. mirabilis* (Wasfi et al., 2020), *S. aureus* (Lister and Horswill, 2014), *Staphylococcus epidermidis (S. epidermidis)* (Walker et al., 2020), *P. aeruginosa* (Walker et al., 2020), and *Streptococcus viridans (S. viridans)* (Veerachamy et al., 2014). Moreover, due to the complex, yet exceptionally humid and nutritional environment, the dental cavity is highly susceptible to biofilm formation (Dewhirst et al., 2010). Bacteria that are responsible for such activity include *Corynebacterium*, *Streptococcus*, *Porphyromonas*, *Haemophilus*/*Aggregatibacter*, *Neisseriaceae*, *Fusobacterium*, *Leptotrichia*, *Capnocytophaga*, and *Actinomyces* (Mark Welch et al., 2016).

Biofilm, acting as ‘protective clothing’, allows the bacteria to thrive well in inhospitable environments such as extreme temperatures and poor nutrient conditions (Harrison et al., 2007; Yin et al., 2019). Due to biofilm’s strong resistance to external stimuli, it is estimated that 65% to 80% of the bacterial infections in the human body are correlated with biofilm (Lebeaux et al., 2013). There are two ways of biofilm infections: through direct infection of body tissue such as via lung infections in cystic fibrosis patients (Lebeaux et al., 2013), and contaminated medical devices or prostheses such as urinary catheters and dentures in patients with urinary tract infections and periodontal infections, respectively (Donlan, 2001; Murakami et al., 2015). Hence, since a broad spectrum of diseases is associated with the presence of biofilm, the ability of bacteria to form biofilms determines its pathogenicity and is of great significance in the course of infection.

## Antimicrobial Compounds on Biofilm Treatment

Among biofilm’s resistance to external pressures, one that presents immense clinical threat is its opposition against antimicrobial activities, chiefly during its mature stage (Wolcott et al., 2010; Wu et al., 2015). Bacteria clustered in biofilm could become up to a thousand times more resistant to antibiotics than the planktonic bacteria cells (Høiby et al., 2011; Wu et al., 2015). Several mechanisms could account for such resistance, as shown in Table 1: (1) Limitation of antibiotic diffusion through EPS matrix. (2) Limitation of antibiotic diffusion via extracellular DNA. (3) Activation of antibiotic-degrading enzymes in the matrix. (4) Horizontal gene transfer. (5) Multispecies interactions.

**TABLE 1:** Biofilm’s antibiotic-resistant approaches and their mechanisms.

Antibiotic-resistant approach	Mechanisms
Limitation of antibiotic diffusion via EPS matrix	The structure of the EPS matrix, notably the exopolysaccharides, provides physical layers of protection against antimicrobial agents by creating permeability barriers that limit its diffusion (Yasuda et al., 1999). Moreover, biofilm EPS contains anionic and cationic molecules that can bind charged antimicrobial agents and accumulate antibacterial molecules up to 25% of its weight (Nadell et al., 2015; Sugano et al., 2016). Hence, the thick layers of EPS matrix may not be responsible for complete antibiotic resistance but provide the time necessary for biofilm to form adaptive phenotypic response to reduce susceptibility (Tseng et al., 2013).
Limitation of antibiotic diffusion via extracellular DNA (eDNA)	The inhibition of bacterial mobility due to the increase of cell density in the biofilm environment creates ideal conditions for direct interaction between conjugative plasmids (eDNA) as well as eDNA and exopolysaccharides (Hu et al., 2012; Rabin et al., 2015). Both interactions lead to the construction of more defined biofilm structures due to the increase in adhesion factors, hence further limiting the diffusion of antimicrobial compounds.
Antibiotic-degrading enzymes in the matrix	Biofilm possesses the ability to collect large amounts of β-lactamase, an antibiotic-degrading enzyme, in the matrix, creating a defensive mechanism that leads to hydrolysis of antibiotics when struck (Schmelcher et al., 2012).
Horizontal gene transfer	The accumulation of bacterial cells within the biofilm facilitates the horizontal gene transfer of the genes responsible for resistance (Bowler et al., 2020).
Multispecies interactions	Interactions between microorganisms that are different species in a biofilm can change the general antimicrobial resistance of the population (Bowler et al., 2020).

Furthermore, the biofilm matrix also protects bacteria from host immune responses. When activating the immune system, biofilm prevents bacteria from neutrophilic phagocytosis by containing the bacteria cells within a thick layer of coating (Yan and Bassler, 2019). Because neutrophils are only capable of engulfing pathogens that are smaller than 10 μm, they would find it impossible to ingest biofilm that could range up to 500 μm in horizontal dimension (Ferkinghoff-Borg and Sams, 2014; Yan and Bassler, 2019).

Despite difficulties combating biofilm, there are potential antimicrobial strategies that have shown to be partly effective. Wolcott et al. have demonstrated the use of sharp debridement techniques to remove the entire biofilm structure at its early stage of formation (Wolcott et al., 2010). While this method of physical scraping led to a decrease in the resistance to gentamicin in biofilm formed on fresh wounds, the regaining of antibiotic resistance after the 24-h therapeutic window as well as the limitation to function only on exposed infected regions have shown that this method is impractical in most cases (Wolcott et al., 2010; Yan and Bassler, 2019). In addition, bacterial enzyme-mediated biofilm dispersal has also gained clinical relevance especially in the eradication of biofilms in the oral cavity, where bacteria may secrete enzymes that downgrade biofilm matrix polymers produced by other pathogens (Kaplan, 2010). Nevertheless, due to the continuous upsurge in the number of multi-drug resistant bacterial strains, clinical and scientific research are in demand for finding alternative strategies to cope with biofilm development (Morris and Cerceo, 2020). Phages, the natural predator of bacteria, could be a solution worth exploring.

## How Phages Combat Biofilm

Phage-based treatment is capable of combating biofilm via several mechanisms using various phage components. Phages are strictly host-specific viruses that infect bacteria and are host-dependent during self-replication. In recent years, with the reduction in new antibiotic discoveries and the increase of antimicrobial resistance (AMR), phage and phage therapy research have gradually made a comeback since the discovery of antibiotics. Thousands of phages have been discovered (Ackermann and Prangishvili, 2012), but its basic structural forms can be categorized into four types: tailed phages, polyhedral phages, filamentous phages, and pleomorphic phages (Ackermann, 2009). The highly specific interaction with the host cell relies upon the receptor-binding protein positioned on the tail fiber of phages (Dams et al., 2019). The antibacterial activity of phage is carried out by two main enzymes—depolymerase and lysins—which are responsible for degrading capsular polysaccharides and peptidoglycan in bacterial cells, respectively (Schmelcher et al., 2012; Yan et al., 2014). The domain of a depolymerase is often displayed at the tip of the phage as tail fibers. On the other hand, lysins are encoded either inside or on the tail of the virion, which cleave the peptidoglycan cell wall from the inside and outside respectively (Sharma et al., 2018).

**FIGURE 3:**
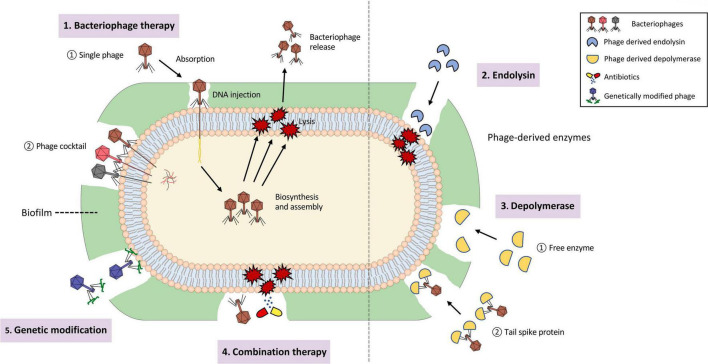
Depiction of biofilm removal using phages and its derived enzymes. (1). Bacteriophage therapy, consisting of single phage therapy and cocktail therapy, that is used for intra- to extracellular degradation of the bacterial structure. (2). Phage-derived endolysin used for extra- to intracellular degradation of the bacterial structure. (3). Phage-derived depolymerase, presented as free enzyme or tail spike protein, that is used for chemical dispersion of the biofilm matrix. (4). Combination therapy using both phages and other antimicrobial compounds, such as antibiotics. (5). Genetically-modified phages that enlarge the host-species interaction range.

Phages are capable of destroying the bacterial hosts and therefore preventing the formation of biofilm (Domingo-Calap and Delgado-Martinez, 2018). Phages could also penetrate existing biofilm and eliminate the biofilm structure with or without killing the resident bacteria (Domingo-Calap and Delgado-Martinez, 2018). In nature, biofilm removal using phages could be categorized into three ways: (i) intra- to extracellular degradation of the bacterial structure. (ii) extra- to intracellular degradation of the bacterial structure. (iii) chemical dispersion of biofilm matrix, notably the EPS structure (Chan and Abedon, 2015). The corresponding modes by which phage-based treatment works are through basic phage therapy, phage-derived lysins, and phage-derived depolymerases (Young, 1992; Schmelcher et al., 2012; Yan et al., 2014). In addition, phages could be structurally engineered or bind with other antimicrobial compounds to produce genetically modified or combination therapies that could enhance the efficacy of eliminating microbial activity. The five ways of biofilm removal mentioned above are depicted in Figure 3. Biofilm is like a “baguazhen,” a fortress-like circle previously mentioned, that is almost insurmountable to common antibiotics. Hence, this paper provides each potential phage-based approach toward this ‘insurmountable fortress’ that will be further discussed in the following sections.

### Phage Therapy

Exploiting phage against bacterial activity can be identified as a form of microorganism-mediated biocontrol, which includes the adoption of the whole organism or solely the organism-derived products as the bacterial antagonist (Chan and Abedon, 2015). On that account, the treatment mediated by the entire phage structure is defined as phage therapy and has shown to be effective in eradicating bacterial biofilm through killing bacteria hosts from “within” (Parasion et al., 2014; Chan and Abedon, 2015; Domingo-Calap and Delgado-Martinez, 2018).

Prior to attaching to the host cell, phages infiltrate biofilm using depolymerases, encoded at the tail structure of the virion, to aid its affinity toward target bacteria (Parasion et al., 2014). Subsequently, the initial interaction between phages and bacteria hosts that leads to viral infection is activated by the receptor-binding protein on the long tail fiber that specifically attaches to the receptors on the surface of the host cell (Islam et al., 2019). Following the irreversible attachment phase are the tail sheesh contraction, tail tube penetration, genome injection, and finally cell lysis (Islam et al., 2019). The intra- to extracellular degradation of the host cell is unique to virulent phages which induce the release of progeny phages from the infected cells at the final stage of the lytic cycle (Cisek et al., 2017). Additionally, this discharge of lytic progeny virions is accompanied by the activation of holins and endolysins, two phage proteins that trigger cell lysis (Cisek et al., 2017). Holins are responsible for piercing the cytoplasmic membrane of the host, while also enabling endolysin to give access to and degrade bacterial peptidoglycan, a major component of the bacterial cell wall (Cisek et al., 2017).

Many studies have been carried out using phage therapy to combat biofilm formation, as shown in Supplementary Table 1. The first case study was conducted by Doolittle et al. in 1995, where Escherichia virus T4, or commonly known as phage T4, was used to eliminate the existing biofilm secreted by *E. coli* (Doolittle et al., 1995). Phage therapy has since then proven to be effective in eradicating biofilm secreted by various bacterial strains, while its studies in clinical settings are also extensive. Amongst them is the eradication of biofilms on the surfaces of medical devices, such as prostheses and catheters (Morris et al., 2019; Cano et al., 2021; Manoharadas et al., 2021). For instance, Morris et al. assessed the anti-biofilm activity of phage toward prosthesis-related infections caused by *S. aureus*. The study mimicked clinical settings by applying a phage cocktail on biofilm-coated three-dimensional-printed titanium that is frequently used in orthopedic implants. The result demonstrated a 3.3-fold reduction in biofilm biomass, as well as a decrease in the thickness and area of the biofilm after 48 h of cocktail exposure (Morris et al., 2019). On the other hand, catheter-associated biofilm clearance may involve with an alternative approach where phage is used as a gel-like coating on the catheter to reduce the bacterial adhesion to the surfaces (Curtin and Donlan, 2006; Maszewska et al., 2018). Furthermore, since the excessive misuse of traditional antibiotics has led to a striking rise in cases related to drug-resistant bacterial infections (World Health Organization [WHO], 2020), phage therapy has recently shifted the focus on eradicating biofilms produced by multi-drug resistant (MDR) bacteria such as MDR *Enterobacter cloacae* (*E. cloacae)* (Jamal et al., 2019), MDR *S. aureus* (Cha et al., 2019), MDR *P. aeruginosa* (Yuan et al., 2019; Adnan et al., 2020), and MDR *Salmonella gallinarum* (*S. gallinarum)*, (Rizzo et al., 2020) to name a few. The diagnostic assays for measuring anti-biofilm activity of phages include fluorescence microscopy (Shahed-Al-Mahmud et al., 2021), LIVE/DEAD BacLight Bacterial Viability Kit (Latka and Drulis-Kawa, 2020), etc. Additionally, biofilm clearance in the oral cavity using phage therapy is also observed in recent years.

#### Dental Biofilm and Related Health Concerns

The oral cavity provides an ideal inhabiting environment for biofilm formation. These biofilms can form on natural dentition and tissues, alongside abiotic surfaces including dental prostheses and implants. Dental biofilms form in a similar process as biofilms in other parts of the body, with planktonic, biofilm, and dispersal phases. Heller et al. detailed a biofilm formation process whereupon immersion in oral cavity fluid, a thin pellicle composed of saliva and glycoproteins is adsorbed onto the tooth surface (Heller et al., 2016). Bacteria aggregate toward this pellicle in a variety of ways, including but not limited to pellicle-bacteria surface molecule interactions and charge-related attachment. As stated above, the main species of bacteria inhabiting oral biofilms can be categorized into nine taxa: *Corynebacterium*, *Streptococcus*, *Porphyromonas*, *Haemophilus*/*Aggregatibacter*, *Neisseriaceae*, *Fusobacterium*, *Leptotrichia*, *Capnocytophaga*, and *Actinomyces*. Mark Welch et al. observed complex microbial coagulation, with *Corynebacterium* acting as a bridge microbe within the biofilm structure. It is known that dental biofilm and related plaque are a direct causes of periodontal diseases such as gingivitis and periodontitis, as well as dental caries (Mark Welch et al., 2016). Certain bacteria within the biofilm, for instance, *Porphyromonas gingivalis (P. gingivalis)*, have shown interactions with stem cells and are linked to several immune diseases including Alzheimer’s disease and rheumatoid arthritis (Kriebel et al., 2018; Olsen and Singhrao, 2020). Many phages have been derived and isolated as of date from several oral pathogenic bacteria including *Fusobacterium*, *Aggregatibacter*, etc. Kabwe et al. (2019) discovered a novel phage FNU1 capable of significantly reducing *Fusobacterium nucleatum* (*F. nucleatum*) biofilm mass by 70%. The study determined that the FNU1 phage was capable of breaking down the biofilm of *F. nucleatum* and lysing the bacteria cells within, thus presenting another viable option in periodontitis treatment.

#### Phage Cocktails Addressing the Limitations of Single Phage Therapy

As seen in Supplementary Table 1, phage therapy entails the preparation of a single phage or a mix of various phages, also known as phage cocktails (Lusiak-Szelachowska et al., 2020). Despite the success of a single type of phage against bacteria activity, the demand for phage cocktails emerged because high specificity in a single phage strain often leads to limitations in identifying the fitting strain. The search for a corresponding strain of phage before treatment can often be problematic especially for emergency cases (Domingo-Calap and Delgado-Martinez, 2018). As a result, the implemented strategy is the preparation of a phage cocktail, which increases the efficiency of such pairing by increasing the range of action (Kifelew et al., 2020). Phage cocktails could also delay the emergence of phage-resistant bacteria by including multiple phages for bacteria to interact with (Domingo-Calap and Delgado-Martinez, 2018). Moreover, different strains of phages could also complement one another by providing the necessary antimicrobial elements that one may be short of. An example of this is demonstrated by Chhibber et al. (2015) who applied phage cocktail therapy against mixed-species biofilm of *K. pneumoniae* and *P. aeruginosa*. The phage cocktail consists of *K. pneumoniae*-specific depolymerase-producing phage KP01K2 and *P. aeruginosa*-specific non-depolymerase-producing phage Pa29. The former phage with degrading enzyme hydrolyzed the outer structure of *K. pneumoniae* to enable the access of Pa29 to *P. aeruginosa* located underneath, hence resulting in a significant reduction of biofilm biomass for both bacteria that may not be possible to eradicate without cocktail approach (Chhibber et al., 2015). All in all, since biofilms are multi-bacterial communities, cocktail therapy is more in demand (O’Toole et al., 2000).

Apart from the limitations in the narrow range of action, growing resistance, and other constraints that could be solved by the use of phage cocktail, phage therapy also presents fundamental concerns (Chan and Abedon, 2015) in its relative usage safety in treating biofilm and other microbial activities. The issue lies in the phage release of inflammatory bacterial proteins, notably endotoxins, due to impure phage preparations (Steele et al., 2020). Poor phage purification may result in having high concentrations of lysed bacteria with inflammatory proteins that instigate the immune system and trigger the inflammatory response (Steele et al., 2020). Nevertheless, researchers such as Luong et al. (2020) have demonstrated the combinational use of centrifugation, microfiltration, and cross-flow ultrafiltration that could remove up to 10^6^ fold of endotoxins in phage preparations, while other researchers have also displayed different effective strategies (Szermer-Olearnik and Boratynski, 2015; Van Belleghem et al., 2017). In order to introduce phage therapy to the masses, the ethical acceptance and social compliance of injecting viruses into the body and potentially treating diseases must also be addressed (Domingo-Calap and Delgado-Martinez, 2018).

### Phage-Derived Enzymes

Phage therapy combats microbial activities largely through two substances: lysin and depolymerase. As these phage-encoded enzymes have also shown effectiveness against biofilm formation, the purification and recombining of the derived enzymes enable alternative choices to withstand challenges related to host specificity and resistance.

#### Lysin

Phage lysins are hydrolytic enzymes and depending on its target bacteria can be labeled as either Gram-positive or Gram-negative lysins. Lysins are generally considered as enzymes produced at the end of the phage lytic replication cycle to cleave the bacterial cell wall from within the cell for release, but can also work externally by assisting bacterial cell penetration of the parental phage. In addition to its phage-related abilities, lysins can degrade the biofilm extracellular polymeric matrix and target the associated bacteria within the matrices.

It is known that lysins generally have the best effect on Gram-positive bacteria, since Gram-negative bacteria have an outer membrane (OM) that restricts lysins from reaching their peptidoglycan cell walls. Despite the challenges faced when combating Gram-negative bacteria, studies have shown promising results, for instance Vasina et al. (2021) found that the four Gram-negative bacteria-targeting endolysins LysAm24, LysAp22, LysECD7, and LysSi3, have high antibacterial activity both *in vitro* and *in vivo*.

In Gram-positive lysins, the C-terminal (cell-binding domain, CBD) is responsible for binding to the cell wall, whereas the N-terminal (enzymatically active domain, EAD) is responsible for catalyzing peptidoglycan hydrolysis. Gram-negative lysins have no use for a CBD and generally utilize a globular configuration with a single EAD to interfere with the bacterial cell wall (Becker et al., 2008). Some Gram-negative lysins have been found to have a modular configuration that consist of an N-terminal peptidoglycan binding domain (PBD) that recognizes the peptidoglycan in Gram-negative bacteria and also the C-terminal EAD similar to Gram-positive lysins (Briers et al., 2009; Walmagh et al., 2012). In addition, some Gram-negative lysins have been found to have a domain, CHAP (cysteine, histidine-dependent amidohydrolase/peptidase), which has the ability to facilitate hydrolysis of the peptidoglycan layer (Sanz-Gaitero et al., 2013; Becker et al., 2015). This ability allows enhancement of its catalytic capabilities, allowing a lysin to be enzymatically active on the peptidoglycan of multiple Gram-negative strains (Walmagh et al., 2013).

Due to the ability of bacteria to form antibiotic-resistant and multidrug-resistant biofilms, lysins used as free enzymes (independently without the parental phage) have shown to be a potential alternative to antibacterial drugs in treating bacterial biofilms. Table 2 demonstrates the antibiofilm lysins trials conducted on a variety of growth sites. As mentioned earlier, biofilm can be a serious threat clinically due to the tendency of it forming in human infections and certain medical devices. As seen in Table 2, *S. aureus*, especially multi-drug resistant *S. aureus* (MRSA) is one of the major target bacteria when it comes to research on lysins, as they are common in clinical settings. In studies from both 2014 and 2017, ClyH and ClyF (both chimeric lysins) have been found to have effect against MRSA (Yang et al., 2014, 2017). Both studies showed a large percentage of biofilm mass reduction when treated with chimeric lysin ClyH or ClyF. In this review, we focus on endolysin (as a free enzyme) activity on combating biofilm and biofilm formation in clinical use.

**TABLE 2:** Anti-biofilm lysin studies.

Author, year	Biofilm-forming bacteria	Phage strain(s)/lysin	Growth site	Results
Landlinger et al., 2021	*Gardnerella*	PM-477 (engineered lysin)	vaginal swabs from BV (bacterial vaginosis) patients	For the majority of the samples, PM-477 demonstrated disruption of biofilm without affecting the remaining vaginal microbiome
Sosa et al., 2020	*S. aureus*	PlySs2	Murine tibial implant	PlySs2 and vancomycin used together *in vivo* reduced the number of CFUs on the surface of implants by 92%
Fursov et al., 2020	*K. pneumoniae*	Prophage/LysECD7	diffusion chambers implanted in outbred rats	Substantial number of viable bacteria in the formed biofilms was disrupted by 50 μg of LysECD7 injected intraperitoneally
Idelevich et al., 2020	*S. aureus*	HY-133 (chimeric lysin)	Vascular graft surface	HY-133 on graft surface-adherent cells was moderate
Schuch et al., 2017	*S. aureus*	Bacterial specific phage/CF-301	Surgical mesh, catheters	In catheters, CF-301 removed all biofilm within 1 h
				Antibiofilm activity of CF-301 was improved in combinations with lysostaphin
				Highly effective for destroying biofilms and biofilm bacteria
Yang et al., 2017	*S. aureus*	187, bacterial specific phage/ClyF (chimeric lysin)	Mouse model of burn wound	ClyF treated burn wounds showed clear degradation of biofilm compared with control group
Yang et al., 2016	*Streptococcus mutans (S. mutans)*	Prophage/ClyR (chimeric lysin)	hydroxyapatite disks	Biofilms formed on hydroxyapatite disks (representing the tooth enamel) reduced by ∼1 log at 50 μg/ml, ∼2 logs at 100 μg/ml, and ∼3 logs at 200 μg/ml
Thandar et al., 2016	*A. baumannii*	P307 and P307SQ-8C (engineered lysins)	polyvinyl chloride (PVC) catheter tubing	After 2 h, approximately 3- and 4-log decreases in CFU/ml were observed with P307 and P307SQ-8C
				After 24 h, an additional ∼1.3-log decrease was observed with P307
Lood et al., 2015	*A. baumannii*	Prophage/PlyF307	Catheters, mouse model	Catheters treated with PlyF307 displayed an approximately 1.6-log-unit decrease in the number of *A. baumannii*
				Mouse models treated with PlyF307 displayed an approximately 2-log-unit decrease in bacterial viability
Yang et al., 2014	*S. aureus*	ClyH (chimeric lysin)	96-well plates	ClyH treated clinical S. aureus isolates showed a > 60% biofilm mass reduction

While the mechanism for antibiofilm disruption by lysins is not fully understood, lysins possess the ability to degrade a substantial amount of the extracellular polymeric matrix of the biofilm. Optimally, lysins or antibiofilm agents in general should have the ability to penetrate biofilm, disrupt its matrix and then combat the bacteria (Rabin et al., 2015). This can be made possible through inhibiting bacterial surface attachment and disruption or destabilization of matured biofilms (Miquel et al., 2016).

The promising future of lysins has a lot to do with its advantages over other antibacterial agents, such as antibiotics or its parental phage. One of the major advantages lysins have over broad-spectrum antibiotics is higher specificity against its target bacteria, which also prevents it from targeting the normal flora (Landlinger et al., 2021). Some other advantages include fast lysis of host cell, synergism when combined with other antibacterials, ability to combat biofilms and lower chance of developing resistance (Schuch et al., 2014; Oliveira et al., 2018). Although lysins are antibacterials with great potential, there are some disadvantages worth mentioning. These include factors like the challenge of finding a suitable drug delivery method, its exceptionally high of a specificity and regulatory body approval, as Murray et al., 2021 has summarized in her review relating to the challenges of lysins used in a clinical setting (Murray et al., 2021).

#### Depolymerase

Depolymerases are a type of enzyme that possesses the ability to degrade the capsular polysaccharides on Gram-negative bacteria, thus providing an entry point for other forms of attack, such as the use of antibacterial drugs. Depolymerases are typically encoded as part of phage structure and several known depolymerases able to function against a range of bacteria species have been identified. Based on the different mechanisms of phage depolymerases, they can be further categorized into two groups: hydrolases and lyases (Knecht et al., 2019). Hydrolases, in contrast to lyases, cleave substrates by hydrolysis—a process that involves the use of a water molecule (Ozaki et al., 2017).

As mentioned above, the main component of biofilm is EPS, which can make up 50 to 90 percent of the total biofilm organic components (Flemming and Wingender, 2010). Hence, these enzymes also hold the ability to inhibit biofilm formation. Phage-derived depolymerases may present two facets of approach toward anti-biofilm treatment, (i) as tail spike protein (TSP), or (ii) as free enzymes. The former approach pertains to purifying the protein present in biofilm-degrading phages and heterologously expressing it as recombinant protein on virion structures. Free depolymerase, on the other hand, presents a certain set of advantages not present when part of a virion (TSP), these include but are not limited to greater molecular stability, reduced chances for resistance formation, and more efficient delivery via diffusion (Chan and Abedon, 2015).

Guo et al. (2017) conducted a trial that analyzed the ORF42 of the vB_EcoM_ECOO78 *E. coli* phage and extracted the depolymerase Dpo42. The enzyme, after further purification, was expressed as a free protein via *E. coli* BL21. The team tested the protein’s ability on *E. coli* and determined that Dpo42 effectively degraded the capsular polysaccharides (CPS) surrounding the *E. coli* as well as prevention of *E. coli* biofilm formation. An advantage of depolymerases may be its ability to degrade the glycocalyx, the main component of both biofilm matrices as well as bacterial capsules (Chan and Abedon, 2015). Moreover, as part of the phage composition, depolymerase also shares advantages including a high specificity toward a specific bacteria species without harming the normal flora, usage in tackling multidrug-resistant (MDR) bacteria, etc. (Lin et al., 2017; Al-Wrafy et al., 2019).

Further research conducted using TSP depolymerase has opened the field to medical device applications. Shahed-Al-Mahmud et al. (2021) utilized tail spike proteins to treat *A. baumannii*-adhered catheters and observed significantly fewer bacteria cells after 4 h of treatment. The study was designed by immersing the catheters into *A. baumannii* culture, thus allowing the formation and growth of biofilm, for seven days and treating with either TSP or PBS control. Zebrafish tested using Ab-54149 with and without the depolymerase demonstrated that after 4 days, TSP-treated zebrafish presented significantly higher survival rates compared to those without TSP treatment. Shahed-Al-Mahmud et al. (2021) proposed that φAB6 TSP would provide potential treatment against MDR *A. baumannii* infections in the near future. Additional anti-biofilm depolymerase trials are demonstrated in Table 3.

**TABLE 3:** Anti-biofilm depolymerase studies.

Author, year	Biofilm-forming bacteria	Phage(s)	Growth site	Results
Shahed-Al-Mahmud et al., 2021	*A. baumannii*	φAB6	96-well microtiter plate	Showed a therapeutic effect in the treatment of *A. baumannii*-induced infections
Chen et al., 2021	*A. baumannii*	vB_AbaM-IME-AB2	96-well plate	Total eradication of human serum bacteria at 50% volume ratio when combination of phage and colistin was applied.
Ku et al., 2021	*P. mirabilis*	KMI8	96 well polystyrene plates	Capable of degrading mono-biofilms of a strain of *Klebsiella michiganensis (K. michiganensis)* that carried the polysaccharide capsule KL70 locus
Rice et al., 2021	*K. michiganensis*	vB_PmiS_PM-CJR	LB agar plates	Characterized a biofilm depolymerase from a *Proteus* phage
Wu et al., 2019	*K. pneumoniae*	SH-KP152226	96-well plate	Specific enzymatic activities in the depolymerization of the K47 capsule Enhance polymyxin activity against *K. pneumoniae* biofilms
Guo et al., 2017	*E. coli*	vB_EcoM_ECOO78	96-well microtiter plate	New potential strategy for preventing *E. coli* biofilm formation

The inclusion of bacterial capsules as a target may lead to a decrease in bacterial virulence and open a pathway for not only phages but also antibiotics as a potential treatment option. It should also be mentioned that depolymerase also presents the ability to be extensively genetically engineered to increase its effectiveness (Topka-Bielecka et al., 2021).

### Combination of Phage and Antimicrobials

The application of phage therapy and virion proteins has displayed immense progress in eradicating biofilm. Yet, some studies have suggested a combinational therapy of phage and other antimicrobial activity, as using phages alone may not be sufficient to eradicate biofilm effectively or permanently. For instance, when Nzakizwanayo et al. (2015) applied phage therapy to eradicate crystalline biofilm formed by *P. mirabili*s on urinary catheters after 10 h of infections, the levels of biofilm formation were significantly reduced but not the number of resident planktonic cells that are available to repeatedly secrete biofilm. While a revised approach was not investigated further by Nzakizwanayo et al. (2015) an advanced elimination of biofilm could be achieved by the combination of phage with other antimicrobial approaches. Doub et al. (2021) presented a successful clinical case where a patient with intractable *S. epidermidis* prosthetic knee infection is recovering without clinical recurrence after being treated with phage therapy and debridement, antibiotics, irrigation, and retention of the prosthesis (DAIR) surgery. This combinational approach by DAIR benefits phage therapy by manually removing the overlying planktonic bacteria as well as parts of the chronic biofilm structure to allow for direct exposure of phage to deep-seated bacteria, thus resulting in complete eradication of biofilm biomass and improvement in clinical therapeutic effect (Doub et al., 2021).

The combination of phages and antibiotics could also address the challenges in the emergence of increasing tolerant bacterial populations against phages. Antibiotics have shown immense success in combating bacterial activity in the last few decades but have gradually unveiled its flaws. As mentioned earlier, the increase in antibiotic-resistant bacteria strains and the inhibition of antibiotic diffusion inside thick EPS matrix led to new opportunities for phage applications. While phages can penetrate the biofilm matrix and the communities within, treatment of biofilms with solely phages could also lead to the emergence of phage-resistant strains and thus, the inability for phages to eradicate biofilm. For instance, Henriksen et al. (2019) examined the effect of repeated phage treatments on *P. aeruginosa* biofilms over time and showed growth of biovolume from 22.24 to 31.07 μm^3^/μm^2^ when treated with phages twice and thrice, respectively. Nonetheless, the biovolume of phage-treated biofilm decreased up to 0.14 μm^3^/μm^2^ after ciprofloxacin was added in Henriksen et al. (2019). Hence, as an increase of phage resistance from bacteria enables its higher sensitivity to antibiotics (Kortright et al., 2019), instead of replacing antibiotics, phages could combine with antibiotics to provide two divergent pressures for resistance prevention (Tagliaferri et al., 2019). This has also been demonstrated in the *K. pneumoniae* biofilm treated with the combinational use of lytic phage KP34 and ciprofloxacin, which led to a significant reduction in the number of resistant variants (Latka and Drulis-Kawa, 2020), as well as in other numerous biofilms (Coulter et al., 2014; Wang et al., 2020).

Recent studies have shed light on the potential of combining depolymerases with other compounds. Chen et al. (2021) identified during a trial, a depolymerase Dpo71 from an *A. baumannii* phage in the heterologous host *E. coli* to combat multidrug-resistant *A. baumannii*. The team concluded after further research that Dpo71 presented the ability to enhance antibiotic activity, specifically colistin, and demonstrated that at 10 μg/ml, Dpo71 enabled a total eradication of human serum bacteria at 50% volume ratio. Dpo71 was also able to inhibit both existing as well as new biofilm formation. Chen et al. further proposed that the potential combination therapy of Dpo71 with colistin could enhance antibiofilm capabilities, therefore, increasing the survival rate of infected patients (Chen et al., 2021). A further list of recent combination therapy is demonstrated in Table 4.

**TABLE 4:** Anti-biofilm combination therapy studies.

Author, year	Biofilm-forming bacteria	Phage strain	Growth site	Results
Chen et al., 2021	*A. baumannii*	vB_AbaM-IME-AB2	96-well plate	Total eradication of human serum bacteria at 50% volume ratio when combination of phage and colistin was applied.
Doub et al., 2021	*S. epidermidis*	PM448	Bacterial site in the intraarticular space of the patient’s prosthetic knee	Combination therapy of phage and debridement, antibiotics, irrigation, and retention of the prosthesis surgery led the patient to recover from recalcitrant prosthetic joint infection by having thorough eradication of biofilm biomass.
Latka and Drulis-Kawa, 2020	*K. pneumoniae*	KP34	96 well plates	Best antibiofilm results where lytic phage KP34 was applied in combination with ciprofloxacin
Kifelew et al., 2020	*S. aureus*	AB-SA01	96-well polystyrene tissue culture plate	Application of phage cocktails led to a significant reduction in bacterial host population within mixed-species biofilm, while combination with tetracycline led to more bacterial population reduction.
Henriksen et al., 2019	*P. aeruginosa*	ATCC 12175-B1, ATCC 14203-B1, ATCC 14205-B1	Flow cells	Single phage treatment led to an 85% to 95% reduction in biofilm’s biovolume.
				Repeated phage treatment enhanced the biovolume of the biofilm after the second and third treatments.
				The combination of phages and ciprofloxacin led to biomass reduction of 6 log units.
				Demonstrated the possibility of bacterial resistance to phages and the effectiveness of combination therapy of phages and antibiotics.
Papadopoulou et al., 2019	*Flavobacterium psychrophilum (F. psychrophilum)*	Fpv-9, Fpv-10	96-well polystyrene microtitre plates	Phage cocktail led to a significant reduction in biofilm biomass after 24-hour exposure
				Anti-biofilm compounds (2-aminoimidazole, emodin, parthenolide, and D-leucine) inhibited biofilm formation for up to 80%.
				Suggesting the higher efficacy of combinational therapy of phage and inhibiting compounds against biofilm.
Chhibber et al., 2015	*K. pneumoniae, P. aeruginosa*	KP01K2, Pa29	Black polycarbonate membrane, 96-well microtiter plates with TSB medium	Led to log-CFU/cm2 biofilm reduction of 3.9 when using KP01K2 for *Klebsiella*, while no significant reduction was observed when using Pa29 for *Pseudomonas*.
				Led to log-CFU/cm2 biofilm reduction of 2.8 when both phages were used.
				Led to complete eradication or log-CFU/cm2 biofilm reduction of 4 when combinational use of KP01K2 and xylitol was used for *Klebsiella* or *Pseudomonas*, respectively.
				Led to log-CFU/cm2 biofilm reduction of 6 when combinational use of KP01K2, Pa29. and xylitol was used for *Pseudomonas*.
				Suggesting the higher efficacy of combinational therapy of phage and xylitol against biofilm
Seth et al., 2013	*S. aureus*	Bacteria-specific phages	Six-millimeter dermal punch wounds in New Zealand rabbit ears	The combination of phage therapy and sharp debridement decreased bacterial biofilm cell counts by a 2-log fold (99% removal).
				Illustrated the effective approach of combining phage therapy and sharp debridement technique.

### Genetically Engineered Phages

The final strategy this paper wishes to present comes via genetic engineering, a recombination process pioneered in 1973 by American biochemists Stanley N. Cohen and Herbert W. Boyer. (Britannica, 2021). The concept presented a multitude of opportunities for scientists to recombine DNA strands to create a phage phenotype suitable for a specific host. In the past 30 years, phages have seen a period of rapid growth, thus leading to different categories and types of recombined, genetically modified phages and phage proteins being developed. Phages can be engineered using several different protocols including but not limited to homologous recombination, phage recombineeringm of electroporation DNA, CRISPR-Cas-based phage engineering, *in vivo* recombineering, etc. (Pires et al., 2016; Chen et al., 2019).

Current research regarding genetically engineered phages can be broadly split into two categories, phage therapy and phage proteins. Lu et al. conducted a trial involving the use of a lysogenic phage M13mp18 with overexpressed lexA3 to increase the antibiotic-induced killing ability toward *E. coli* (Lu and Collins, 2009). The team demonstrated that the lexA3-producing phage together with ofloxacin, an antibiotic, significantly increased the antibacterial effect against wild-type *E. coli* EMG2. Edgar et al. conducted a trial by means of gene delivery via homologous recombination (Edgar et al., 2012). Phages carrying the homologously recombinant genes rpsL (sensitive to streptomycin) and gyrA (sensitive to nalidixic acid) were administered to induced antibiotic-resistant E. coli and a significant MIC decrease was later observed. Lu et al. also engineered a T7 phage expressing an *Aggregatibacter actinomycetemcomitans (A. actinomycetemcomitans)* biofilm-degrading enzyme dispersion B (Lu and Collins, 2007). The team discovered that the T7 phage was effective against *E. coli* TG1 biofilms by a log4.5 reduction.

Phage proteins, especially lysins previously discussed, also present the potential to be genetically engineered to maximize and broaden their effectiveness. There has been growing interest in modified lysins with novel characteristics, especially engineered lysins and chimeric lysins when combating biofilm. Engineered lysins are novel lysins with customized features created by swapping its modular domains, for instance, artificial lysins (artilysins) that are created by combining a natural lysin fragment with peptides or other proteins (Rodriguez-Rubio et al., 2016; Schirmeier et al., 2018). Chimeric lysins (also known as chimeolysins) are formed by switching the domains of the natural lysin, such as the cell wall binding domains (CBDs) and the catalytic domains (CDs) (Huang et al., 2020; Li et al., 2021). Engineered lysins used in countering biofilm are well represented in anti-biofilm lysin trials as shown in Table 2. Landlinger et al., 2021 conducted a study that found an engineered lysin, PM-477 to be active against *Gardnerella* biofilms (Landlinger et al., 2021). Lysin PM-477 was created by recombining EADs and CBDs, testing various combinations on bacterial strains to find a final combination that is the most efficient.

## Concluding Remarks

There are five main approaches to countering the biofilm matrix. (1) Phage therapy that entails the use of the whole organism, which eradicates bacterial biofilm through killing bacteria hosts from “within” via the initial penetration of the matrix by depolymerase followed by the lytic cycle. (2) Phage-derived depolymerase which could be used as a TSP or free enzyme and works by degrading the EPS, CPS, and glycocalyx. (3) Phage-derived endolysins that infiltrate the EPS structure and combat the local bacteria externally. (4) Combination therapy that is associated with the application of phage and other antimicrobial compounds for more complete eradication of both the matrix and the dormant bacteria, as well as decrease in resistance toward phages. (5) Genetically engineered phages enlarge the host-species interaction range by modifying the proteins involved in the phage attachment and/or insertion. The future of phage therapy focuses on expanding the scope of phage and its derived enzymes which could be achieved by further exploration of: (i) combinational therapy with phage and antibiotics; (ii) genetically engineered phages; (iii) genetically engineered proteins such as artilysins, chimeolysins that overcome the limitations allowing endolysins to target gram-negative bacteria.

## Author Contributions

CC, XY, and WG wrote this article. CG provided some comments. XG, QL, and YZ conducted the research and revised the manuscript. All authors contributed to the article and approved the submitted version.

## Conflict of Interest

The authors declare that the research was conducted in the absence of any commercial or financial relationships that could be construed as a potential conflict of interest.

## Publisher’s Note

All claims expressed in this article are solely those of the authors and do not necessarily represent those of their affiliated organizations, or those of the publisher, the editors and the reviewers. Any product that may be evaluated in this article, or claim that may be made by its manufacturer, is not guaranteed or endorsed by the publisher.
