# Supplementary material for: Bacteriophage-Mediated Control of Biofilm: A Promising New Dawn for the Future
Source: Front Microbiol. 2022 Apr 4;13:825828. doi: 10.3389/fmicb.2022.825828 (PMC9048899; doi:10.3389/fmicb.2022.825828)
Supplement: Supplementary file 1 [file Table_1.DOCX]

Supplementary Material

# Supplementary Tables

Table S1: List of anti-biofilm bacteriophage therapy trials

| Author, year | Biofilm-forming bacteria | Phage strain | Growth site | Results |
| --- | --- | --- | --- | --- |
| (Cano et al., 2021) | *K. pneumoniae* | KpJH46Φ2 | Bacterial site in the right lower extremity of the patient; 96-well polystyrene microtitre plates | - In vivo anti-biofilm activity showed patients recovering from swelling, pain, and limited range of motion of right lower extremity. The 34-week follow-up showed no sign of symptom recurrence. - In vitro anti-biofilm activity showed a trend in biofilm biomass reduction after 22 hours of its exposure to phage. |
| (Manoharadas et al., 2021) | *S. aureus* | ɸ44AHJD | Sterile glass coverslips | - Eliminated existing biofilm on smooth glass surface after 72h of infection. - Revealed the possibility of removing biofilm on a clinically relevant smooth glass surface. |
|  | *E. coli* | ɸX174 |  |  |
| (D'Andrea et al., 2020) | *E. faecalis* | vB_EfaH_EF1TV | 96-well microtiter plates with TSB | - Biofilm reduction observed in confocal laser scanning microscopy. |
| (Szymczak et al., 2020) | *B. bronchiseptica* | vB_BbrP_BB8 | 96-well polystyrene microtiter plates with BHI medium | - Led to 75%, 71%, and 59% of biofilm biomass reduction for phage concentrations of 10^7^, 10^5^, and 10^3^ PFU/mL, respectively. |
| (Rizzo et al., 2020) | MDR S. gallinarum | UPF_BP1 and UPF_BP2 | 96-well polystyrene plates with TSB medium | - 85% of the biofilm strains were susceptible to at least one phage, while among those are 74% lysed by both phages. |
| (Adnan et al., 2020) | MDR *P. aeruginosa* | MA-1 | 96-well plates with TSB medium | - Led to 2.1 fold, 2,5 fold, and 3.2 fold reductions in biomass in 24, 48, and 72h biofilm, respectively. |
| (Morris et al., 2019) | *S. aureus* | StaPh_1, StaPh_3, StaPh_4, StaPh_11, StaPh_16 | 3D-printed, porous titanium cylindrical scaffolds. | - Led to log-CFU/cm2 biomass reduction of 6.8 to 6.2 after exposure to StaPhage cocktail, while a decrease in the thickness and area of the biofilm was also seen after 48 hours of cocktail exposure. - Demonstrated the possibility of removing biofilm on clinically relevant orthopedic material. |
| (Gupta et al., 2019) | *E. coli, S. aureus, P. aeruginosa* | Bacteria-specific phages | Human chronic non-healing wound | - Led to significant improvement in wound healing among 20 patients after 3 to 5 doses of topical bacteriophage therapy, with 7 of the patients reaching complete healing after 21 days. |
| (Yuan et al., 2019) | MDR *P. aeruginosa* | vB_PaeM_LS1 | Coverslips placed over a 6-well plate with LB medium | - Eliminated existing biofilm |
| (Cha et al., 2019) | MDR *S. aureus* | CSA13 | 96‐well polystyrene plate with TSB | - Biofilms formed by MSSA and MRSA were reduced by 78% and 93% in its biomass after 24h of infection. |
| (Jamal et al., 2019) | MDR *E. cloacae* | MJ2 | Stainless steel plates with TSB | - Led to 2.8-. 3-, and 3.5-log reductions in biomass in 24, 72, and 120h biofilm, respectively. |
| (Gu et al., 2019) | MDR uropathogenic *E. coli* | vB_EcoP-EG1 | 96-well plates with LB medium | - Biofilms formed by MG1655 and 390G7 were reduced by 60% and 50% in its biomass after 24h of infection, respectively. |
| (Kwiatek et al., 2017) | *P. aeruginosa* | MAG1 and MAG4 | 96-well microtiter plates | - Led to 60% and 80% reduction in biofilm biomass when using MAG1 and MAG4 after 8h of infection, respectively. - MAG1 selected less for phage-resistant clones - The efficacy of the phage cocktail was similar to that of MAG4 alone |
| (Shafique et al., 2017) | *P. aeruginosa* | JHP | 96-well polystyrene microtitre plates | - Phage treatment before biofilm formation decreased bacterial cell counts for up to 9 logs (>95% removal) |
| (Alves et al., 2016) | *P. aeruginosa* | DL 52, DL 54, DL 60, DL 62, DL 64, DL 68 | 96-well polystyrene microtitre plates | - Static and dynamic biofilms were eradicated almost completely after 4h and 48h of phage cocktail infections, respectively. - Applied phage cocktail therapy on the dispersion of *P. aeruginosa* biofilm. |
| (Lehman and Donlan, 2015) | *P. aeruginosa, P. mirabilis* | Bacteria-specific phages | Artificial urine medium | - Led to log-CFU/cm2 biofilm reduction of 4 and 2 for *P. aeruginosa* and *P. mirabilis* biofilm after 48h of cocktail pretreatment, respectively. - Demonstrated the significance of phage cocktail pretreatment on reducing mixed-species biofilm formed on a urinary catheter. |
| (Danis-Wlodarczyk et al., 2015) | *P. aeruginosa* | KTN28 | 96-well microtiter plates with TSB medium | - Led to a significant reduction of biofilm biomass in 24h, 48h, and 72h biofilm |
| (Jamal et al., 2015) | MDR *K.pneumoniae* | Z | 96-well microtitre plates | - Led to 2 fold, and 3 fold reductions in biomass in 24 and 48h biofilm, respectively. |
| (Nzakizwanayo et al., 2015) | *P. mirabilis* | Bacteria-specific Phages | Bladder model (double-walled glass chamber) | - Led to a significant reduction in crystalline biofilm formation but not the number of planktonic cells. |
| (Alves et al., 2014) | *S. aureus* | DRA88, K | 96-well polystyrene tissue culture microplates | - Biofilms formed separately by 15981, MRSA 252, H325 were either eradicated or disrupted by more than 50% after 48h of infection at MOI of 10. - Applied phage cocktail therapy on the dispersion of *S. aureus* biofilm. |
| (Yele et al., 2012) | *A. baumannii* | AB7-IBB1 | Abiotic surface (polystyrene);  biotic surface (human embryonic kidney 293 cell line) | - 75% of the biofilms were removed from the abiotic surface at an MOI of 105 with 102 CFU/well. - 50% of the biofilms were inhibited from the biotic surfaces at an MOI of 103 with 102 CFU/well. |
| (Kelly et al., 2012) | *S. aureus* | K, and six modified derivatives (K.W73365, K.ST22ISA67, K.ST39ISA108, K.MS811, K.ST30ISA58, K.M255039) | 96-well microtiter plate with Trypticase soy broth (TSB) medium | - Led to a significant reduction in biofilm biomass after 72h of cocktail infection - Initial contact of the phage cocktail led to complete inhibition of biofilm formation over a 48h period with no indication of phage resistance |
| (Kim et al., 2012) | *P. aeruginosa, S. aureus, S. epidermidis, Staphylococcus hominis (S. hominis)* | PA1Ø | 96-well microtiter plate | - The reductions of numbers in mixed bacteria cells in phage-treated biofilms were evident, while electron microscopy analysis also displayed biofilm removal activities - Presented the board bactericidal spectrum of such phage and the possibility of using a single phage strain to treat mixed infections caused by multiple bacteria |
| (Son et al., 2010) | *S. aureus* | SAP-2 | 96-well polystyrene microplate well | - While phage showed its ability to erase biofilm, phage-derived endolysin SAL-2 expressed a broader spectrum of activity. - Shed light on the separate use of phage-derived enzymes. |
| (Curtin and Donlan, 2006) | *S. epidermidis* | 456 | Lubri-sil all-silicone 16 French Foley catheters | - Led to log-CFU/cm2 biofilm reduction of 4.47 and 2.34 with and without supplemental divalent cations, respectively. - Pretreatment of phage on the catheter surface prevented biofilm formation. |
| (Doolittle et al., 1995) | *E. coli* | T4 | Polyvinyl chloride coupons placed in modified Robbins devices | - Eliminated existing biofilm |

Reference:

Adnan, M., Ali Shah, M.R., Jamal, M., Jalil, F., Andleeb, S., Nawaz, M.A., et al. (2020). Isolation and characterization of bacteriophage to control multidrug-resistant Pseudomonas aeruginosa planktonic cells and biofilm. *Biologicals* 63**,** 89-96. doi: 10.1016/j.biologicals.2019.10.003.

Alves, D.R., Gaudion, A., Bean, J.E., Perez Esteban, P., Arnot, T.C., Harper, D.R., et al. (2014). Combined use of bacteriophage K and a novel bacteriophage to reduce Staphylococcus aureus biofilm formation. *Appl Environ Microbiol* 80(21)**,** 6694-6703. doi: 10.1128/AEM.01789-14.

Alves, D.R., Perez-Esteban, P., Kot, W., Bean, J.E., Arnot, T., Hansen, L.H., et al. (2016). A novel bacteriophage cocktail reduces and disperses Pseudomonas aeruginosa biofilms under static and flow conditions. *Microb Biotechnol* 9(1)**,** 61-74. doi: 10.1111/1751-7915.12316.

Cano, E.J., Caflisch, K.M., Bollyky, P.L., Van Belleghem, J.D., Patel, R., Fackler, J., et al. (2021). Phage Therapy for Limb-threatening Prosthetic Knee Klebsiella pneumoniae Infection: Case Report and In Vitro Characterization of Anti-biofilm Activity. *Clin Infect Dis* 73(1)**,** e144-e151. doi: 10.1093/cid/ciaa705.

Cha, Y., Chun, J., Son, B., and Ryu, S. (2019). Characterization and Genome Analysis of Staphylococcus aureus Podovirus CSA13 and Its Anti-Biofilm Capacity. *Viruses* 11(1). doi: 10.3390/v11010054.

Curtin, J.J., and Donlan, R.M. (2006). Using bacteriophages to reduce formation of catheter-associated biofilms by Staphylococcus epidermidis. *Antimicrob Agents Chemother* 50(4)**,** 1268-1275. doi: 10.1128/AAC.50.4.1268-1275.2006.

D'Andrea, M.M., Frezza, D., Romano, E., Marmo, P., Henrici De Angelis, L., Perini, N., et al. (2020). The lytic bacteriophage vB_EfaH_EF1TV, a new member of the Herelleviridae family, disrupts biofilm produced by Enterococcus faecalis clinical strains. *J Glob Antimicrob Resist* 21**,** 68-75. doi: 10.1016/j.jgar.2019.10.019.

Danis-Wlodarczyk, K., Olszak, T., Arabski, M., Wasik, S., Majkowska-Skrobek, G., Augustyniak, D., et al. (2015). Characterization of the Newly Isolated Lytic Bacteriophages KTN6 and KT28 and Their Efficacy against Pseudomonas aeruginosa Biofilm. *PLoS One* 10(5)**,** e0127603. doi: 10.1371/journal.pone.0127603.

Doolittle, M.M., Cooney, J.J., and Caldwell, D.E. (1995). Lytic infection of Escherichia coli biofilms by bacteriophage T4. *Can J Microbiol* 41(1)**,** 12-18. doi: 10.1139/m95-002.

Gu, Y., Xu, Y., Xu, J., Yu, X., Huang, X., Liu, G., et al. (2019). Identification of novel bacteriophage vB_EcoP-EG1 with lytic activity against planktonic and biofilm forms of uropathogenic Escherichia coli. *Appl Microbiol Biotechnol* 103(1)**,** 315-326. doi: 10.1007/s00253-018-9471-x.

Gupta, P., Singh, H.S., Shukla, V.K., Nath, G., and Bhartiya, S.K. (2019). Bacteriophage Therapy of Chronic Nonhealing Wound: Clinical Study. *Int J Low Extrem Wounds* 18(2)**,** 171-175. doi: 10.1177/1534734619835115.

Jamal, M., Andleeb, S., Jalil, F., Imran, M., Nawaz, M.A., Hussain, T., et al. (2019). Isolation, characterization and efficacy of phage MJ2 against biofilm forming multi-drug resistant Enterobacter cloacae. *Folia Microbiol (Praha)* 64(1)**,** 101-111. doi: 10.1007/s12223-018-0636-x.

Jamal, M., Hussain, T., Das, C.R., and Andleeb, S. (2015). Characterization of Siphoviridae phage Z and studying its efficacy against multidrug-resistant Klebsiella pneumoniae planktonic cells and biofilm. *J Med Microbiol* 64(Pt 4)**,** 454-462. doi: 10.1099/jmm.0.000040.

Kelly, D., McAuliffe, O., Ross, R.P., and Coffey, A. (2012). Prevention of Staphylococcus aureus biofilm formation and reduction in established biofilm density using a combination of phage K and modified derivatives. *Lett Appl Microbiol* 54(4)**,** 286-291. doi: 10.1111/j.1472-765X.2012.03205.x.

Kim, S., Rahman, M., Seol, S.Y., Yoon, S.S., and Kim, J. (2012). Pseudomonas aeruginosa bacteriophage PA1O requires type IV pili for infection and shows broad bactericidal and biofilm removal activities. *Appl Environ Microbiol* 78(17)**,** 6380-6385. doi: 10.1128/AEM.00648-12.

Kwiatek, M., Parasion, S., Rutyna, P., Mizak, L., Gryko, R., Niemcewicz, M., et al. (2017). Isolation of bacteriophages and their application to control Pseudomonas aeruginosa in planktonic and biofilm models. *Res Microbiol* 168(3)**,** 194-207. doi: 10.1016/j.resmic.2016.10.009.

Lehman, S.M., and Donlan, R.M. (2015). Bacteriophage-mediated control of a two-species biofilm formed by microorganisms causing catheter-associated urinary tract infections in an in vitro urinary catheter model. *Antimicrob Agents Chemother* 59(2)**,** 1127-1137. doi: 10.1128/AAC.03786-14.

Manoharadas, S., Altaf, M., Alrefaei, A.F., Hussain, S.A., Devasia, R.M., Badjah Hadj, A.Y.M., et al. (2021). Microscopic analysis of the inhibition of staphylococcal biofilm formation by Escherichia coli and the disruption of preformed staphylococcal biofilm by bacteriophage. *Microsc Res Tech* 84(7)**,** 1513-1521. doi: 10.1002/jemt.23707.

Morris, J., Kelly, N., Elliott, L., Grant, A., Wilkinson, M., Hazratwala, K., et al. (2019). Evaluation of Bacteriophage Anti-Biofilm Activity for Potential Control of Orthopedic Implant-Related Infections Caused by Staphylococcus aureus. *Surg Infect (Larchmt)* 20(1)**,** 16-24. doi: 10.1089/sur.2018.135.

Nzakizwanayo, J., Hanin, A., Alves, D.R., McCutcheon, B., Dedi, C., Salvage, J., et al. (2015). Bacteriophage Can Prevent Encrustation and Blockage of Urinary Catheters by Proteus mirabilis. *Antimicrob Agents Chemother* 60(3)**,** 1530-1536. doi: 10.1128/aac.02685-15.

Rizzo, N.N., Pottker, E.S., Webber, B., Borges, K.A., Duarte, S.C., Levandowski, R., et al. (2020). Effect of two lytic bacteriophages against multidrug-resistant and biofilm-forming Salmonella Gallinarum from poultry. *Br Poult Sci* 61(6)**,** 640-645. doi: 10.1080/00071668.2020.1805724.

Shafique, M., Alvi, I.A., Abbas, Z., and Ur Rehman, S. (2017). Assessment of biofilm removal capacity of a broad host range bacteriophage JHP against Pseudomonas aeruginosa. *APMIS* 125(6)**,** 579-584. doi: 10.1111/apm.12691.

Son, J.S., Lee, S.J., Jun, S.Y., Yoon, S.J., Kang, S.H., Paik, H.R., et al. (2010). Antibacterial and biofilm removal activity of a podoviridae Staphylococcus aureus bacteriophage SAP-2 and a derived recombinant cell-wall-degrading enzyme. *Appl Microbiol Biotechnol* 86(5)**,** 1439-1449. doi: 10.1007/s00253-009-2386-9.

Szymczak, M., Grygorcewicz, B., Karczewska-Golec, J., Decewicz, P., Pankowski, J.A., Orszagh-Szturo, H., et al. (2020). Characterization of a Unique Bordetella bronchiseptica vB_BbrP_BB8 Bacteriophage and Its Application as an Antibacterial Agent. *Int J Mol Sci* 21(4). doi: 10.3390/ijms21041403.

Yele, A.B., Thawal, N.D., Sahu, P.K., and Chopade, B.A. (2012). Novel lytic bacteriophage AB7-IBB1 of Acinetobacter baumannii: isolation, characterization and its effect on biofilm. *Arch Virol* 157(8)**,** 1441-1450. doi: 10.1007/s00705-012-1320-0.

Yuan, Y., Qu, K., Tan, D., Li, X., Wang, L., Cong, C., et al. (2019). Isolation and characterization of a bacteriophage and its potential to disrupt multi-drug resistant Pseudomonas aeruginosa biofilms. *Microb Pathog* 128**,** 329-336. doi: 10.1016/j.micpath.2019.01.032.
